# Accuracy of the “Timed Up and Go” Test for Predicting Low Muscle Mass in a Preoperative Prehabilitation Program for Colorectal Cancer

**DOI:** 10.3390/jcm14062088

**Published:** 2025-03-19

**Authors:** Leticia Pérez-Santiago, Luisa Paola Garzón-Hernández, José Martín-Arévalo, Vicente Pla-Martí, David Moro-Valdezate, David Casado-Rodrigo, Marina Riera-Cardona, Noelia Tarazona, Bianca Tabita Muresan, Ning Yun Wu Xiong, Alejandro Espí-Macías, Stephanie García-Botello

**Affiliations:** 1 Colorectal Surgery Unit, Department of General and Digestive Surgery, Hospital Clinico Universitario, INCLIVA Biomedical Research Institute, Avenida Blasco Ibáñez Nº 17, 46010 Valencia, Spain; balita1393@hotmail.com (L.P.G.-H.); martin_jose@gva.es (J.M.-A.); vplamarti@yahoo.es (V.P.-M.); davidmoro78@gmail.com (D.M.-V.); dcasador@hotmail.com (D.C.-R.); marina.riera.cardona@gmail.com (M.R.-C.); alejandro.espi@uv.es (A.E.-M.); stephanie.garcia@uv.es (S.G.-B.); 2 Department of Surgery, University of Valencia, Avenida Blasco Ibañez, Nº 13, 46010 Valencia, Spain; 3 Department of Medical Oncology, INCLIVA Biomedical Research Institute, University of Valencia, Avenida Blasco Ibañez, Nº 13, 46010 Valencia, Spain; noetalla@incliva.es; 4 Centro de Investigación Biomédica en Red de Cáncer (CIBERONC), Instituto de Salud Carlos III, 28029 Madrid, Spain; 5 Department of Physiotherapy, Faculty of Health Sciences, European University of Valencia (UE), Avenida Blasco Ibañez, Nº 13, 46010 Valencia, Spain; bianca.muresan90@gmail.com; 6 Department of Endocrinology and Nutrition, Hospital Clinico Universitario, 46010 Valencia, Spain; nywx.224@hotmail.com

**Keywords:** colorectal cancer, timed up and go, sarcopenia, muscle mass

## Abstract

**Background:** Preoperative sarcopenia is associated with increased morbidity and mortality in patients undergoing colorectal cancer (CRC) surgery. The assessment of muscle mass is crucial in identifying at-risk patients, but standard imaging methods like computed tomography (CT) scans require significant resources. Functional tests, such as the Timed Up and Go (TUG) test, may serve as simple and effective alternatives for sarcopenia screening. **Objective:** To evaluate the accuracy of the TUG test in predicting preoperative sarcopenia in patients scheduled for CRC surgery. **Methods:** A prospective observational study was conducted at a tertiary colorectal unit from January 2022 to June 2023. Patients underwent a prehabilitation assessment, including the TUG test, four weeks before surgery. Sarcopenia was diagnosed based on reduced muscle mass measured at the third lumbar vertebra on CT images. Statistical analyses included the sensitivity, specificity, and overall accuracy of the TUG test in predicting sarcopenia. **Results:** The study included 199 CRC patients (58.3% male, mean age 71.76 ± 10.42 years). Sarcopenia was present in 48.7% of patients. The mean TUG test length was 12.52 ± 7.95 s. A TUG test time of ≥10.19 s predicted sarcopenia with 70.1% sensitivity, 75.5% specificity, and an overall accuracy of 72.9% (95% CI = 0.660–0.790). **Conclusions:** The TUG test is a reliable, simple, and non-invasive tool for identifying sarcopenia in patients scheduled for colorectal cancer surgery, reducing reliance on CT scans. Early detection allows for timely interventions, improving surgical outcomes and overall patient prognosis.

## 1. Introduction

Colorectal cancer (CRC) is the third most prevalent cancer worldwide and has the second highest mortality [1]. Surgical treatment is potentially curative in most cases and according to published series, surgery is associated with a 20–30% complication rate [2,3].

Disease-related malnutrition (DRM), when present in patients with CRC, is a factor that negatively impacts survival and responses to both oncological and surgical treatment [4]. It is estimated that between 15 and 40% of cancer patients present malnourished, increasing the incidence up to 60–80% in advanced disease cases. The most severe form of malnourishment in cancer patients is cancer cachexia, which is accompanied by a progressive loss of muscle mass [5]. The neoplasm directly affects nutritional status, causing significant muscle mass depletion through catabolic processes and increasing the risk of malnourishment, leading to a decrease in the functional capacity of patients [6]. Recent European guidelines on preoperative assessment emphasize the importance of identifying high-risk patients and modifiable risk factors before elective surgery, which is particularly relevant in malnourished cancer patients [7].

Currently, sarcopenia has been associated with worse outcomes in cancer patients, decreasing overall survival, increasing postoperative morbidity, and negatively influencing quality of life [8]. Its relationship with CRC has been documented in several studies, with a high prevalence ranging between 40 and 60%, making preoperative diagnosis crucial [9]. According to an international group which gathered to define sarcopenia in cancer patients, it is a fundamental component of cancer cachexia and a critical element in the comprehensive evaluation of cancer patients.

In the last consensus of the European Working Group on Sarcopenia in Older People (EWGSOP2), published in 2018, sarcopenia was defined as being probable when low muscle strength is detected. It is confirmed when there is low muscle quantity or quality, and it should be considered severe if there is also a low physical performance [10]. Sarcopenia is more common in the elderly due to age-related muscle changes, with muscle mass decreasing by 1–2% per year and muscle strength by 1.5–5% per year after the age of 50 [11]. However, other factors such as associated illnesses, inactivity, and malnourishment contribute to and exacerbate muscle deterioration, leading to a more rapid onset. Based on its etiology, sarcopenia can be classified as primary (due to ageing) or secondary (resulting from pathogenic mechanisms such as inflammatory and neoplastic processes) [9]. In oncological patients, preoperative sarcopenia has been independently associated with poor prognosis and is a recognized risk factor for postoperative complications in oncological surgery [12,13,14]. Other negative outcomes related to sarcopenia include prolonged hospital stays and, consequently, higher healthcare costs [13].

Given these implications, recognizing preoperative sarcopenia in patients with CRC is imperative for offering prevention and treatment strategies, optimizing surgical outcomes, reducing postoperative complications, and improving overall survival and quality of life. Three primary parameters must be considered during the assessment of sarcopenia: muscle strength, muscle quantity, and physical performance status [10].

Functional tests play a crucial role in evaluating the physical performance component of sarcopenia [10]. They provide valuable insights into the real-world functional status of patients, complementing muscle mass measurements. One commonly used test is the Timed Up and Go (TUG) test, which assesses mobility, balance, and functional capacity. This test is simple, quick to administer, and reproducible, making it a useful tool for the primary assessment of patients [15]. Functional tests such as TUG allow clinicians to identify patients with impaired muscle function who may not yet show significant muscle mass loss on imaging studies. Other functional assessments, such as gait speed tests and grip strength measurements, have also been validated for evaluating sarcopenia in different clinical settings [16].

Beyond functional testing, several imaging modalities are available for muscle mass quantification. Techniques such as muscle ultrasound, bioimpedance analysis, and computed tomography (CT) scans provide precise muscle measurements. Among them, the preoperative evaluation of muscle area in the region of the third lumbar vertebra (L3) by CT scan allows for the calculation of the skeletal muscle index (SMI) and the identification of presarcopenia, characterized by low muscle mass without an impact on muscle strength [17,18]. However, imaging methods are often resource-intensive, requiring specialized equipment, and may not be readily available in all clinical settings, underscoring the importance of functional tests as a complementary assessment strategy.

The goal of this study is to analyze whether the TUG functional test is a valid measure in assessing low muscle mass compared to muscle mass measurement via CT scan in patients with CRC undergoing surgical intervention.

## 2. Materials and Methods

We conducted a prospective, observational, single-centre study from January 2022 to June 2023 at the Hospital Clínico Universitario of Valencia, Spain.

### 2.1. Patient Selection

#### 2.1.1. Inclusion Criteria

Patients were eligible for inclusion if they met the following criteria:

- Aged 18 years or older.
- Provided written informed consent to participate.
- Undergoing elective surgery with colon or rectum resection for CRC.
- Pathological stage I-III.
- Attended a prehabilitation consultation at least four weeks before surgery.
- Underwent the “Timed Up and Go” (TUG) test.
- Underwent a computed tomography (CT) scan for presarcopenia assessment.

#### 2.1.2. Exclusion Criteria

- Emergency surgery.
- Pathological stage IV CRC.
- Missing essential clinical data (e.g., weight, height).
- Contraindication for CT scan.
- Patients with severe mobility impairments preventing the performance of the TUG test, defined as those unable to stand up from a chair without assistance or walk a minimum distance of 3 m.
- Cognitive or language barriers preventing the comprehension of TUG test instructions.

### 2.2. Prehabilitation Consultation

Patients were scheduled for an outpatient prehabilitation consultation managed by a surgeon trained in perioperative protocols, following an interdisciplinary Enhanced Recovery After Surgery (ERAS) approach. This consultation, conducted at least four weeks before the planned surgical intervention, included the following:

- Anthropometric measurements: Body weight (kg) and height (m) were recorded to calculate Body Mass Index (BMI, kg/m^2^), categorized as follows for older adults:
  ○ Underweight: <18.5 kg/m^2^;
  ○ Normal weight: 18.5–24.9 kg/m^2^;
  ○ Overweight: 25.0–29.9 kg/m^2^;
  ○ Obese: ≥30.0 kg/m^2^.
- Prehabilitation counselling: Patients received education on trimodal prehabilitation, which included nutrition, physical exercise, and psychological strategies for anxiety control.
- Functional and body composition assessments:
  ○ TUG test

**Figure 1:**
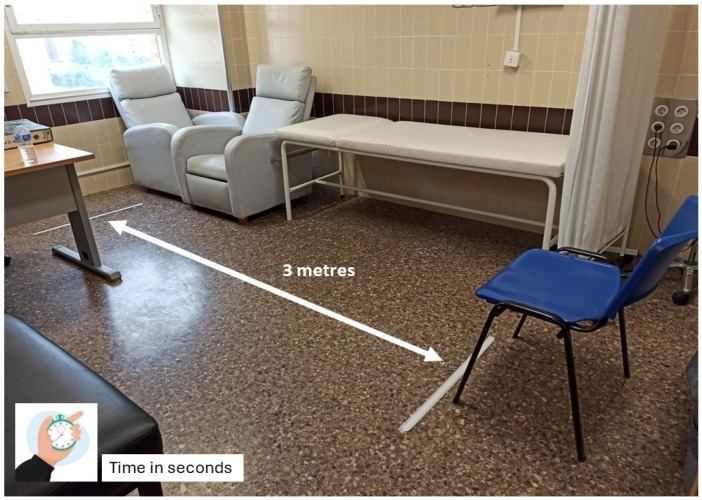
Timed Up and Go test performed in the outpatient clinic. The patient must rise from a chair, walk 3 metres, make a 180 degree turn, return to the chair, and sit down.

The TUG test evaluates functional mobility and fall risk [14]. The test involves the following (Figure 1):

1. The patient sitting in a chair with armrests, positioned against a wall to ensure consistency.
2. Standing up without external assistance, following a verbal instruction.
3. Walking a distance of 3 m on a flat, unobstructed surface at a comfortable, self-selected pace.
4. Returning to the starting position and sitting back down.

The time taken to complete the test is recorded in seconds using a standardized digital stopwatch. To ensure consistency and reduce inter-observer variability, the same trained evaluator conducted all TUG tests throughout the study. The tests were performed at a consistent time of day, mostly in the mornings, in a quiet environment with standard instructions: “When I say ‘go’, stand up from the chair, walk at your normal pace to the marked line, turn around, walk back, and sit down again.” The patients were given a single practice trial before the timed assessment. To minimize potential bias, the assessors conducting the TUG test were not involved in the evaluation of muscle mass using CT scans and were blinded to other clinical data.

EWGSOP2 suggests that scores ≤10 s (s) indicate normal mobility, while times exceeding this threshold suggest the need for further evaluation and intervention.

○ Determination of muscle mass using computed tomography (CT scan)

We perform the calculation of muscle mass on the CT scan at the level of the third lumbar vertebra, using the National Institutes of Health (NIH) Image J^®^ software (64-bit version of Java) [17]. This CT scan is ordered for tumour surgery treatment and conducted four weeks prior to the surgical intervention. Images are analyzed using Hounsfield Unit (HU) thresholds, these being −29 to +150 for skeletal muscle mass (SMM). In patients requiring neoadjuvant therapy, the calculation is performed at the end of the treatment.

**Figure 2:**
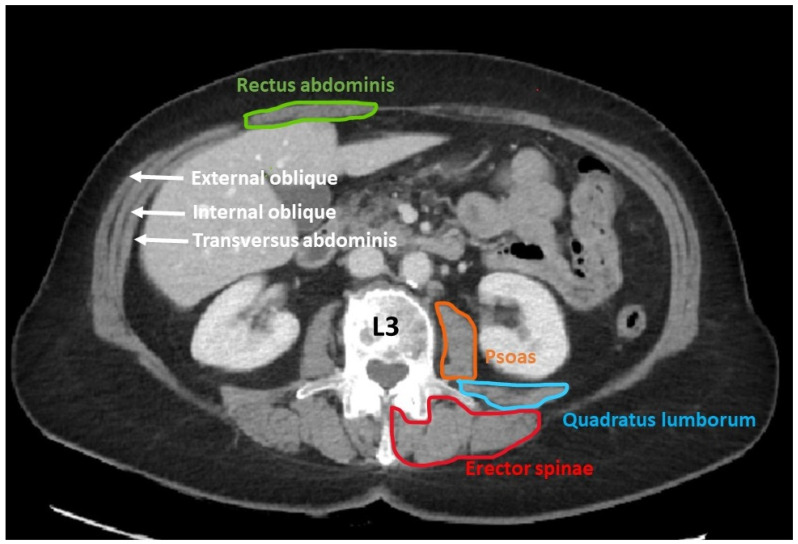
Example of axial CT section at the L3 level where the different muscle groups used to calculate muscle mass are visualized. This region contains different muscles including the psoas (orange colour) and paraspinal muscles, transversus abdominis (white), quadratus lumborum (blue), erector spinae (red), external and internal obliques of the abdomen (white), and rectus abdominis (green). The chosen image is one with both transverse processes clearly visible, normally in the middle of the L3.

The muscle groups measured at the L3 level include the psoas, quadratus lumborum, erector spinae, transversus abdominis, external and internal obliques, and rectus abdominis (Figure 2).

The anonymized images are exported via USB in DICOM format. Through the software, the waist circumference (WC), skeletal muscle mass (SMM), and SMI are calculated.

**Figure 3:**
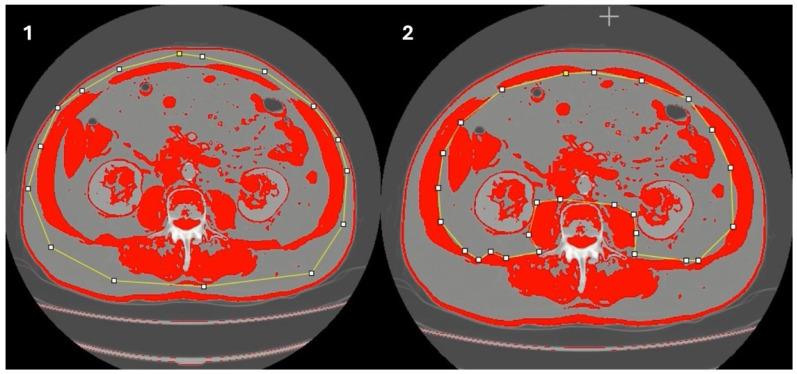
An example of how SMI is measured with the NIH^®^ software. First, the outer perimeters of the abdominal muscles are traced (**1**), followed by the inner muscle perimeter (**2**).

First, the outer perimeters of the abdominal muscles are traced (Figure 3(1)), followed by the inner muscle perimeter (Figure 3(2)). We calculate the SMM: Measurement 1 (outer area)–Measurement 2 (inner area) divided by 100. And finally, the SMI is calculated: SMM/height^2^.

The calculation of muscle mass parameters is performed by the same trained evaluator throughout the study to maintain consistency and reduce measurement variability. Additionally, a second expert reviewed the calculations to ensure accuracy. We emphasize again that the evaluator responsible for muscle mass assessment is blinded to the TUG test results and is not the same person who conducted the functional assessment, minimizing potential bias between functional and imaging-based measurements.

The currently established cutoff points for diagnosing reduced muscle mass are as follows [18]: SMI < 43 cm^2^/m^2^ in males with BMI < 25 kg/m^2^; SMI < 53 cm^2^/m^2^ in males with BMI ≥ 25 kg/m^2^; and SMI < 41 cm^2^/m^2^ in females.

An example of how SMI is measured with the NIH^®^ software is shown in Figure 3.

### 2.3. Statistical Analysis

The study variables included demographic factors such as age, sex, comorbidities, and tumour location. Outcome variables included tests performed during the prehabilitation consultation, such as the result of the TUG test and muscle mass calculated by CT scan.

A descriptive analysis was performed, expressing qualitative variables using absolute numbers and percentages, and continuous quantitative variables using the mean and standard deviation in the case of normal distribution, and the median and interquartile range in cases with non-normal distribution. The Shapiro–Wilk test was used to assess variable distribution.

A logistic regression model was developed for the prediction of preoperative sarcopenia. The model was constructed using the lrm function from the rms package in R (version 4.4.1). In order to ensure the reliability and robustness of the model, an internal validation was carried out using bootstrap resampling with 200 iterations (B = 200). The validation function was used to assess model performance metrics, including corrected c-index, calibration slope, and Nagelkerke’s R^2^. In addition, the calibration of the model was assessed using the calibrate function with 1000 bootstrap replicates (B = 1000). These methods reduced the risk of overfitting and ensured reliable clinical applicability by providing a rigorous assessment of the model’s predictive accuracy and generalisability to new data.

An ROC (Receiver Operating Characteristic) curve of the sarcopenia prediction model with a 95% confidence interval was performed and the area under the curve was calculated. The optimal cutoff of the values obtained from the preoperative sarcopenia test was calculated by finding the point with the maximum sensitivity and specificity on the ROC curve.

A *p* value < 0.05 was considered statistically significant. Statistical analysis was carried out with SPSS^®^ Statistics Version 25 for Windows and R^®^ Version 4.2.1 for Windows with rms and vcd libraries.

### 2.4. Ethical Considerations

All CT images were used for the surgical cancer treatment (without extra radiation for body composition). This study was approved by the Ethics Committee of Hospital Clínico Universitario of Valencia in 2019. Moreover, all images and data collection were used in an anonymized mode and patients gave their written consent before study entry.

## 3. Results

### Patient Characteristics

Two hundred and fifty-seven patients diagnosed with CRC scheduled for surgical resection of the primary tumour were included. A total of 58 patients were excluded from the present study after the exclusion criteria were applied.

In total, 116 (58.7%) males were evaluated, with a mean age of 71.76 (range = 51) years old. The most common location of colorectal cancer (CRC) was the colon (75.4%), which also included neoplasms of the upper rectum.

A total of 97 patients (48.7%) had a low muscle mass, as calculated by CT scan, with a mean SMI of 47.99 ± 10.37 cm^2^/m^2^. Sarcopenia was equally present in men and women (58 (50%) males and 41 (50%) women). According to the TUG measurements, 85 (42.7%) patients presented with a low performance status. Most of the patients were overweight before surgery (66.3%), with a mean BMI of 27.46 ± 4.71 kg/m^2^. The clinical characteristics of the patients are shown in Table 1.

**Figure 4:**
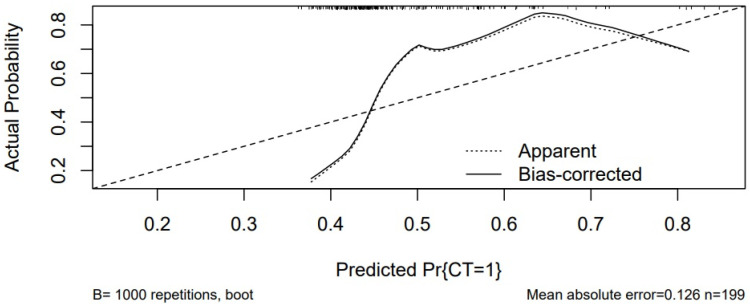
Calibration of the model for sarcopenia prediction based on the test.

**Figure 5:**
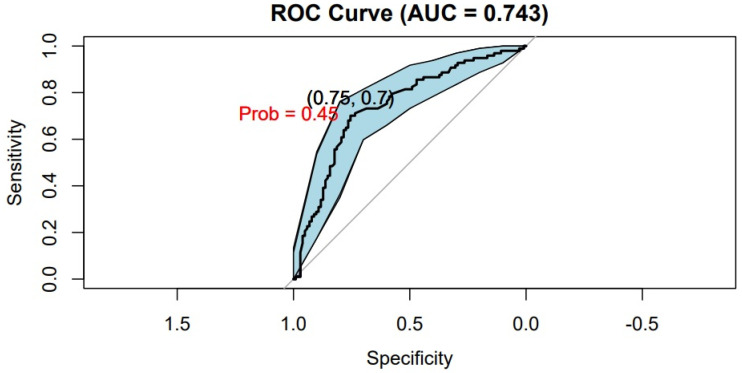
Accuracy of the Timed Up and Go test to predict sarcopenia.

The prediction model for sarcopenia based on TUG was created and internal validation with bootstrap was performed (Figure 4). The AUC of the model based on the TUG test for predicting low muscle mass calculated by CT was 0.743 (95% CI = 0.67–0.81; *p* value ≤ 0.001) In the ROC curve analysis, we found that this test had a sensitivity of 70.1% and a specificity of 75.49% in predicting sarcopenia (Figure 5).

The optimal cutoff point for predicting low muscle mass was a TUG test time greater than 10.19 s, as determined by the ROC curve (Figure 5).

**Figure 6:**
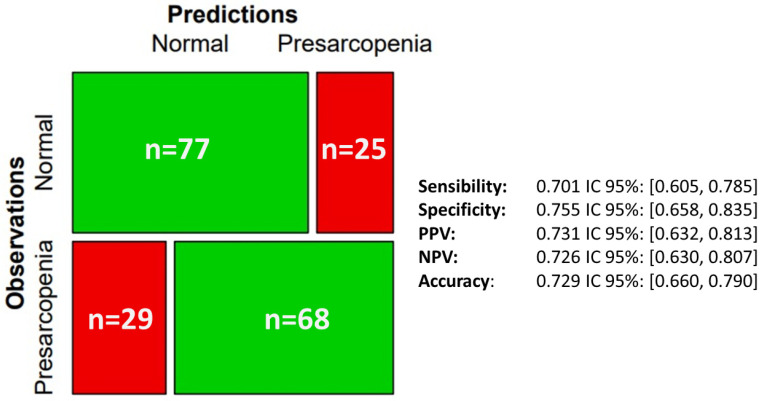
Mosaic graphic of the accuracy of the model predictions.

The confusion matrix (Figure 6) comparing the predicted classifications of “normal” and “presarcopenia” with the actual observations is shown in the mosaic plot. The model showed a sensitivity of 0.701 (95% CI: 0.605, 0.785). This indicates its ability to correctly identify 70.1% of the true presarcopenia cases. The specificity was 0.755 (95% CI: 0.658, 0.835), reflecting the model’s accuracy in identifying 75.5% of true normal cases. The positive predictive value (PPV) was 0.731 (95% CI: 0.632, 0.813), indicating that 73.1% of the cases predicted as “presarcopenia” were true positives. The negative predictive value (NPV) was 0.726 (95% CI: 0.630, 0.807). This means that 72.6% of the cases predicted to be “normal” were in fact not sarcopenic. The overall accuracy of the model, reflecting its overall ability to correctly classify 72.9% of cases, was 0.729 (95% CI: 0.660, 0.790). In distinguishing between “normal” and “presarcopenia” cases, these metrics suggest a balanced performance with a moderate predictive ability.

In the intergroup comparison according to the obtained cutoff point, patients with a TUG test greater than 10.19 s were older (77.51 ± 8.00 vs. 67.47 ± 9.96; *p*: <0.001) and had a lower SMM (119.78 ± 30.59 vs. 137.41 ± 33.33; *p*: <0.001) and SMI (44.77 ± 9.37 vs. 50.39 ± 10.46; *p*: <0.001). Height, weight, and body mass index (BMI) did not show a significant relationship with muscle functionality (Table 2).

## 4. Discussion

This study demonstrated that the TUG test predicted low muscle mass with moderate accuracy in patients diagnosed with CRC and evaluated in a prehabilitation outpatient clinic. In the literature, there is only one study which has assessed the ability of the TUG test to predict the presence of presarcopenia, and it was in hospitalized elderly patients without oncological disease [11]. Therefore, our study is the first to evaluate it in younger patients with oncological disease and in an outpatient setting.

It is important to remember that the TUG test is a physical performance test that measures risk of falls, and is used in many studies in elderly individuals [17]. In a study published in 2014, the association between low muscle mass and poor physical performance in older women was demonstrated [19]. However, it has also been evaluated in children and adolescents; in a 2013 review, the TUG test was shown to be a good tool for assessing functional mobility in pediatrics [20]. One reason the TUG test is related to sarcopenia is due to changes in muscle composition, secondary either to ageing or associated pathologies, which can occur in parallel with a reduction in muscle strength.

Several studies have evaluated the TUG test for fall risk, but there is scant literature analyzing its relationship with low muscle mass, which is a relevant factor in oncological and postoperative outcomes in patients with CRC [21,22,23]. The cutoff point of the TUG test in the current study to predict sarcopenia was greater than 10.19 s, which is very similar to the cutoff point in the study by Martínez et al., which was 10.85 s [11]. When the TUG test is <10 s, it should be interpreted as normal; between 10 and 20 s, patients are considered frail with a risk of falling, and >20 s, patients need assistance [17].

In our study, the TUG test had a direct relationship with elderly patients, those with low muscle mass, and those with a low skeletal muscle mass index. However, weight, height, and BMI were not directly related to having a TUG test greater than 10.19 s and, therefore, with the diagnosis of presarcopenia. With these results, we emphasize the importance of assessing body composition, which includes, among other measures, a patient’s amount of muscle mass. In 2021, a study was published in which Brown et al. corroborated these findings and highlighted that body weight masks clinically significant changes in skeletal muscle, suggesting preoperative body composition measurement using CT images and de-emphasizing the weight and BMI of patients [24].

When comparing performance metrics with other screening tools, such as the Short Physical Performance Battery (SPPB) or the 4 m gait speed test, the TUG test offers a simpler and faster alternative. While the TUG test demonstrated moderate predictive value with an AUC of 0.743, future studies could enhance its utility by evaluating it alongside these other tools to establish a more comprehensive assessment protocol.

All patients diagnosed with CRC should undergo preoperative imaging to assess distant disease, usually through a CT scan, which we use to extract the image and calculate muscle mass. Despite having preoperative imaging tests available, calculating muscle mass using CT requires training and time, which cannot be employed during the care of our patients in outpatient consultations. The time spent diagnosing the amount of muscle mass can be reduced by performing the TUG test, as it is a very simple tool that any healthcare professional can perform and takes less than half a minute to complete. Additionally, since it does not require additional tests or irradiation, it can be repeated as often as necessary, both for preoperative diagnosis and to assess improvement if measures to prevent sarcopenia are applied, such as protein supplements and/or supervised physical exercise programmes to increase muscle mass before surgery.

Furthermore, this study can support the use of this test in diagnosing reduced muscle mass in patients with a benign colorectal pathology who will undergo major surgery, such as chronic diverticulitis or inflammatory bowel disease. In this patient group, imaging tests are often outdated and do not provide a current value of the muscle mass status; however, the TUG test can be performed in the clinic, offering a much more accurate and updated diagnosis.

Expanding on the clinical implications, the findings of this study could support a shift in current practice by incorporating the TUG test more widely as a routine screening tool for sarcopenia in preoperative patients with CRC. Given its ease of use and quick administration time, the TUG test could provide a practical method to identify at-risk patients earlier and initiate prehabilitation interventions, potentially improving surgical outcomes.

The cost-effectiveness of the TUG test also warrants discussion. Unlike imaging-based methods that require equipment, trained personnel, and time, the TUG test is a low-cost and scalable option. The potential savings from avoiding more expensive and resource-intensive diagnostics could be significant, especially in outpatient settings where rapid assessments are needed.

Future research in this area is recommended to explore more advanced approaches and emerging technologies. For example, incorporating AI-assisted technology could add significant value. Some recent studies highlight the use of advanced imaging techniques for body composition analysis, which could enhance the accuracy of muscle mass measurement using computed tomography (CT) scans, as proposed in this study [25].

Additionally, for physical performance assessment, research like that of Rahmati M. et al. provides a broader context on the importance of physical performance measurements, including tests like the Timed Up and Go (TUG) test. This aligns well with the present study’s focus on functional assessment and could enrich the interpretation of the obtained results [26].

Overall, adopting more advanced tools and expanding the theoretical framework with complementary studies could help to validate and optimize the use of the TUG test as a clinical assessment tool, always considering the balance between convenience and diagnostic accuracy.

It is important to note that the moderate predictive value of the TUG test (AUC of 0.743) indicates that this test may be more appropriate as a screening tool rather than a definitive diagnostic test. Considering the balance between convenience and diagnostic accuracy, future studies should focus on validating the TUG test alongside other assessment methods to enhance its clinical utility and ensure the accurate identification of sarcopenia in preoperative patients.

The prevalence of low muscle mass in our sample was almost 45%, emphasizing the importance of early diagnosis. While the TUG test showed moderate validity in predicting sarcopenia, this study was prospective, it is essential to acknowledge that this was a single-centre study.

Our study population was predominantly elderly (mean age of 71.76 years) and overweight (mean BMI of 27.46). This demographic profile may limit the applicability of the findings to younger patients or those with different body compositions, highlighting the need for further research to draw clearer conclusions in diverse populations.

Additionally, another significant limitation is that the TUG test can only be administered to individuals with good mobility. In patients with mobility impairments due to stroke, osteoarthritis, or other pathologies, muscle mass measurement should be performed using standard body composition study mechanisms. Addressing these limitations in future research could improve the generalizability of the findings and support more accurate clinical assessments across a broader range of patient populations.

## 5. Conclusions

The TUG test is a good predictor of low muscle mass in patients diagnosed with CRC. It is a simple and quick test that does not require special equipment and allows for the screening of sarcopenia during the patient’s first general surgeon visit.

## Figures and Tables

**Table 1:** Baseline characteristics of the patients included in the study.

Variable	Value
Age (years)	71.76 (51)
WC (cm)	105.85 ± 14.50
SMM (cm^2^)	129.88 ± 33.28
SMI (cm^2^/m^2^)	47.99 ± 10.37
Weight (kg)	74.13 ± 14.69
Height (m)	1.64 ± 0.90
BMI (kg/m^2^)	27.46 ± 4.71
TUG test (s)	12.52 ± 7.95

Waist circumference (WC); Skeletal muscle mass (SMM); Skeletal muscle index (SMI), body mass index (BMI); Timed up and go (TUG) test.

**Table 2:** Intergroup comparison based on the point of maximum precision in the TUG test.

Variable	Timed Get Up and Go ≥ 10.19 s	Timed Get Up and Go < 10.19 s	*p*-Valour
Age (years)	77.51 ± 8,00	67.47 ± 9.96	<0.001
SMM (cm^2^)	119.78 ± 30.59	137.41 ± 33.33	<0.001
SMI (cm^2^/m^2^)	44.77 ± 9.37	50.39 ± 10.46	<0.001
Height (kg)	74.1 ± 14.70	74.15 ± 14.74	0.98
Weight (m)	1.62 ± 0.09	1.64 ± 0.09	0.10
BMI (kg/m^2^)	27.81 ± 4.62	27.19 ± 4.77	0.36

Skeletal muscle mass (SMM); Body mass index (BMI).

## Data Availability

The original contributions presented in this study are included in the article. Further inquiries can be directed to the corresponding author.
